# Bacterial Species Involved in Venous Leg Ulcer Infections and Their Sensitivity to Antibiotherapy—An Alarm Signal Regarding the Seriousness of Chronic Venous Insufficiency C6 Stage and Its Need for Prompt Treatment

**DOI:** 10.3390/microorganisms12030472

**Published:** 2024-02-26

**Authors:** Sergiu-Ciprian Matei, Cristina Stefania Dumitru, Ayman Mohamed Fakhry, Nenad Ilijevski, Slobodan Pešić, Jovan Petrović, Zorin Petrişor Crăiniceanu, Marius-Sorin Murariu, Sorin Olariu

**Affiliations:** 1 Abdominal Surgery and Phlebology Research Center, Victor Babeș University of Medicine and Pharmacy, 300041 Timisoara, Romania; matei.sergiu@umft.ro (S.-C.M.); murariu.marius@umft.ro (M.-S.M.); srnolariu@yahoo.com (S.O.); 2 1st Surgical Department, Pius Brînzeu Emergency County Hospital, 300736 Timisoara, Romania; 3 Department of Microscopic Morphology/Histology, Angiogenesis Research Center, Victor Babeș University of Medicine and Pharmacy, 300041 Timisoara, Romania; 4 Department of Vascular Surgery, Military Academy, Cairo 1000, Egypt; ayman_vasc@live.com; 5 Vascular Surgery Clinic, Institute for Cardiovascular Diseases “Dedinje”, 11040 Belgrade, Serbia; nilijevskidr@gmail.com (N.I.); spesic90@gmail.com (S.P.); jovanpetrovic1997@gmail.com (J.P.); 6 Faculty of Medicine, University of Belgrade, 11120 Belgrade, Serbia; 7 Department of Plastic and Reconstructive Surgery, Victor Babeș University of Medicine and Pharmacy, 300041 Timisoara, Romania; zcrainiceanu@gmail.com

**Keywords:** venous leg ulcer, *Pseudomonas aeruginosa*, bacteria, antibiotic sensitivity, chronic venous insufficiency

## Abstract

Background: Venous leg ulcers (VLUs) are a common chronic wound condition susceptible to infection by various bacterial species. Understanding bacterial presence and antibiotic sensitivity is crucial for effective treatment. Methodsː Medical records of 60 patients diagnosed with the C6 chronic venous insufficiency stage were analyzed retrospectively. The patients were divided into an active recurrent VLU group (33 cases) and a first-onset active VLU group (27 cases). Bacterial identification, antibiotic sensitivity, and laboratory markers were assessed. Resultsː *Pseudomonas aeruginosa* was the most prevalent bacterial species in both the study (72.72%) and control (37.03%) groups, along with other common bacteria such as *Proteus mirabilis*, *Enterococcus* sp., *Staphylococcus aureus*, *Acinetobacter baumannii*, *Klebsiella* spp., and *Escherichia coli*. Furthermore, uncommon bacteria, including *Providencia rettgeri*, *Group B Streptococcus*, and *Salmonella Paratyphi B*, and a fungal infection with *Candida albicans*, were identified only in the study group, while *Morganella morganii* was found exclusively in the control group. *Pseudomonas aeruginosa* showed significant sensitivity to several antibiotics, particularly Amikacin and Meropenem. Nonspecific laboratory markers, such as CRP, fibrinogen, ESR, WBC, CK, neutrophils, and lymphocytes, revealed statistically significant differences between groups, indicating their potential as biomarkers for monitoring recurrent VLUs. Conclusionsː These results highlight the need for comprehensive diagnostic approaches to effectively manage VLU infections and improve patient outcomes. Further research is warranted to explore factors influencing the presence of uncommon bacteria and to develop targeted interventions for VLU management.

## 1. Introduction

Of the many presentations across the clinical spectrum of chronic venous disease, venous leg ulceration can be considered amongst the most important [1]. Venous leg ulcers are one of the most common types of wounds that appear on the lower part of the legs. [2]. The prevalence is estimated at 1% in the general population, rising to 4% for patients who are over eighty years old [3]. Importantly, with the prevalence increasing in the elderly and significant negative effects on quality of life due to disability, social isolation, and psychosocial burden, venous leg ulceration will continue to present an important challenge, particularly in light of the expected increase in ageing and increasingly obese population. Venous leg ulcers represent a significant burden on patients, and for healthcare systems and national economies too, constituting a costly medical problem with a high toll on worldwide healthcare systems [4,5]. VLU treatment accounts for an annual expenditure of 1–2% of the national health budget, equating to over USD 2.5 billion in the United States and GBP 300–600 million in the United Kingdom [1].

The pathophysiology of VLU is complex and multifactorial, involving factors including genetic predisposition, environmental factors, hormones, endothelial dysfunction, inflammatory cells and molecules and activation on the endothelium and vein wall, and disturbances in the balance of cytokines and matrix metalloproteinases [6].

Chronic wounds of the lower extremities are susceptible to bacterial invasion [7], with Gram-positive (genus *Staphylococcus*, genus *Corynebacterium*) or Gram-negative (genus *Pseudomonas*) aerobic bacteria being usually involved in VLU infection [8]. Healing could be delayed in many cases due to a persistent inflammatory response and infection, and it may take months to years to heal [2,7]. The treatment of chronic leg ulcers remains one of the most challenging issues for phlebologists [9]. Guidelines from The Society for Vascular Surgery and the American Venous Forum consensus recommend compression therapy as the primary treatment to aid the healing of venous ulceration (grade 1B recommendation) [10,11]. Once the ulceration is closed, venous reflux ablation is required to prevent recurrence. However, the role of several drugs in treating the wound is also considered. Signs of infection are the main reason for the use of oral antibiotics. When an ulcer fails to heal, the use of oral aspirin and pentoxifylline can be considered as adjuncts [12], especially in the case of mixed etiology ulcers (venous and arterial), wherein the arterial flow is more precarious and the antibiotic concentration that reaches the infection site is reduced.

This retrospective study followed the results of bacteriological cultures, the sensitivity to antibiotics, and changes in clinical and inflammatory parameters in two groups of patients in the C6 (active venous ulcer) and C6r (recurrent active venous ulcer) stages of chronic venous disease. The aim of this study was to highlight the changes that appear concerning the type and antibiotic sensitivity of the bacterial species among recurrent VLUs, as well as to point out the particular changes that occurred in the biochemical parameters along with the chronic evolution of the disease.

## 2. Materials and Methods

*Patients and data collection*. This study retrospectively analyzed the medical records of the patients treated in the Phlebology Department, Emergency County Hospital Timisoara, Romania between January 2016 and December 2022. A total of 815 patients with phlebological issues were treated in this period of time. Among them, we identified 82 patients diagnosed with first-onset active (C6 stage venous insufficiency, according to the CEAP classification) or active recurrent (C6r stage venous insufficiency, according to the CEAP classification) VLUs. These patients were included in the initial study group. The following parameters were analyzed: age, gender, native environment (urban/rural), wound closure period, microbiological samples, sensitivity to anti-infective chemotherapy and antibiotics (the sensitivity was categorized as resistant, sensitive, or intermediately sensitive), blood cell count and inflammatory markers (total number of red and white blood cells, i.e., RBCs and WBCs; the percentages of neutrophils and lymphocytes; fibrinogen; erythrocyte sedimentation rate, or ESR; C-reactive protein, or CRP), and additional biochemical parameters (creatine kinase (CK), creatine kinase myocardial band (CK-MB), glycemia, hemoglobin A1C-Hba1c, creatinine, uremia).

Patients for whom the data could not be obtained were excluded, and 60 patients were included in the study. These patients were subsequently divided into those with active recurrent VLUs (C6r, 33 cases) and, as control, active venous ulcers of the first-onset type (C6, 27 cases). Five (15.15%) cases from the recurrent VLU group and two (7.4%) cases from the first-onset active VLU group presented polymicrobial infections; thus, a total of 38 antibiograms were analyzed for the study group, and 29 for the control group.

Specimen collection, microbiological techniques, culture conditions, and culture media: All the patients were tested in the hospital on the same day they were admitted. Consequently, each patient’s wound secretion was tested for bacterial and fungal (considering the risk of fungal superinfection after bacterial colonization in chronic wounds) infections, with swab samples being collected. All the samples were transported to the hospital’s laboratory in less than 30 min and were processed according to the standard protocols. For each bacterial culture, the samples were inoculated on solid media, with the following types of culture media being used: blood and chocolate agars, pseudosel agar (cetrimide agar), Chapman’s agar (salt–mannitol agar), and MacConkey’s agar. Sabouraud dextrose agar culture media was used to cultivate dermatophytes and other types of fungi. Using the standard disc diffusion method, antimicrobial susceptibility testing was performed for all the positive bacterial cultures. According to the standard protocols, the sensitivity to the following anti-infective chemotherapy and antibiotics was tested: Amikacin (AK), Ceftazidime (CAZ), Ceftriaxone (CRO), Cefazolin (CZ), Cefepime (CEF), Cefuroxime (CXM), Cefoperazone (CFP), Ciprofloxacin (CIP), Imipenem (IPM), Gentamicin (GM,) Levofloxacin (LVX), Meropenem (MERO), Piperacillin (PIP), Piperacillin+tazobactam (TZP), Streptomycin (S), Ticarcillin (TIC), Tobramycin (TM), Ampicillin (AM), Oxacillin (OX), Amoxicillin + clavulanic acid (AMC), Trimethoprim/sulfamethoxazole (SXT), Clindamycin (CM), Erythromycin (E), and Vancomycin (VA). Additionally, the sensitivity to the following antifungal medications was tested in case of fungal infection: Econazole (ECN), Miconazole (MIC), Amphotericin B (AMB), Fluconazole (FCZ), Nystatin (NYS), Voriconazole (VOR), and 5-Fluorocytosine (5-FC). All the microbiological results presented and analyzed in our study represent the data provided by the laboratory.

*Data analyses.* Statistical analyses were completed using MedCalc^®^ Statistical Software, version 20.118 (MedCalc Software Ltd., Ostend, Belgium; 2022). The Mann–Whitney test and the Chi-square test were employed for the analysis of non-parametric data, ensuring accurate comparisons across the different variables and groups under study. The normality of the distribution of continuous variables was verified using the Kolmogorov–Smirnov test. For continuous parametric data, comparison was performed utilizing Student’s *t*-test. All statistical tests were conducted with a significance level set at two-sided *p* < 0.05, clearly associating each testing method with the specific metrics or variables compared, such as clinico-demographic characteristics, and microbiological data. This comprehensive approach allowed for a robust analysis of the differences between the first-onset active VLU group and the recurrent VLU group, providing a solid foundation for our findings.

*Ethical approval statement.* The ethics committee of Emergency County Hospital Pius Brîınzeu, Timișoara, RO approved this study (REC number: 401/24.07.2023).

## 3. Results

Demographic and clinical characteristics of patients from both groups are presented in Table 1. The period required until granulation tissue appeared and the epithelialization of the skin ulceration took place varied between the groups. The wound-closure mean period was 52.09 days (range: 17–79 days) for the study group and 24.48 days for the control group (range: 14–49 days), with a significant statistical difference between the groups being noted (*p* = 0.031).

**Figure 1:**
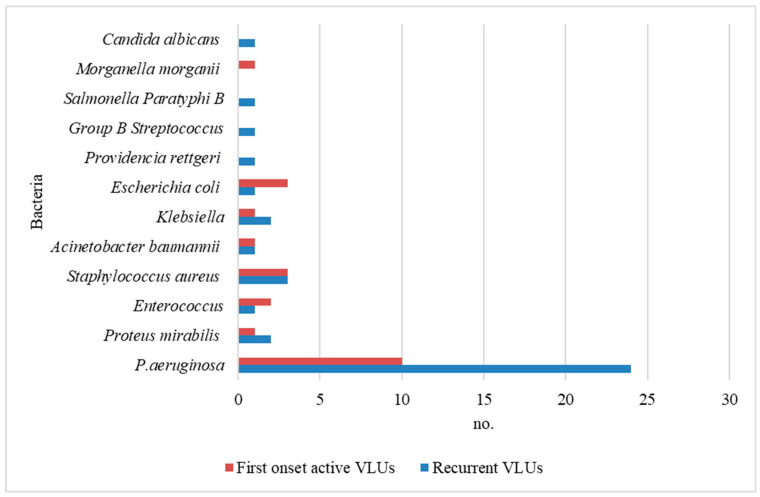
Type of bacterial species present in both groups.

In five cases from the recurrent VLU group, we encountered more than one type of bacterial species in the same wound (with four cases with two types of bacterial species identified and one case with three types of bacterial species identified). An association of two types of bacterial species in the same wound was observed only in two cases from the first-onset VLU group. Also, in seven cases from the first-onset active VLU group, no microorganism was isolated in the culture (sterile bacteriological culture), like in the recurrent VLU group, where at least one type of bacterial species was encountered in the wound. Thus, this study identified 37 types of bacterial species and one type of fungus species in recurrent VLU infections and 22 types of bacterial species in the first-onset active VLU group. Antibiogram (sensitivity to anti-infective chemotherapy and antibiotics) results were analyzed comparatively. Among the bacterial species identified, *P. aeruginosa*, *Proteus mirabilis*, *Enterococcus* sp., *Staphylococcus aureus,* and others were found in both groups. In addition, *Providencia rettgeri*, *Group B Streptococcus*, *Salmonella Paratyphi B,* and *Candida albicans* were identified in the recurrent VLU group while *Morganella morganii* was found only in the first-onset active VLU group (Figure 1). *P. aeruginosa* was the most prevalent type of bacterial species isolated in both the groups (recurrent VLU group—72%; first VLU group—37%).

**Figure 2:**
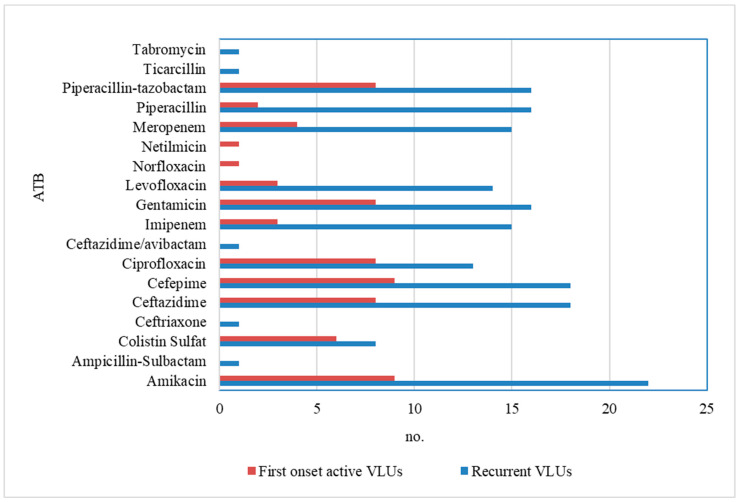
Antibiotic susceptibility (sensitive) of *P. aeruginosa* compared in both groups.

The following is a summary of the antibiotic sensitivity results for the most common type of bacterial species present in both groups (Figure 2). *P. aeruginosa* showed sensitivity to several antibiotics (Ceftazidime, Cefepime, Ciprofloxacin, Imipenem, Gentamicin, Levofloxacin, Piperacillin, and Piperacillin/tazobactam), particularly Amikacin and Meropenem.

In addition, intermediate sensitivity (10% of cases in the recurrent VLU group and 9% in the first-onset active VLU group) was observed in Streptomycin, Cefepime, Imipenem, and Levofloxacin. Antibiotic resistance in 12% of cases in the recurrent VLU group and 9% in the first-onset active VLU group was for Ceftriaxone, Cefazolin, Cefepime, Ciprofloxacin, Levofloxacin, Meropenem, Piperacillin, and Ticarcillin (Table 2).

The study assessed the correlation between various laboratory tests and their association with the condition under investigation (Table 3).

## 4. Discussion

Chronic venous insufficiency is a common primary care problem associated with significant morbidity [13], with the C6 stage according to the CEAP classification (active venous leg ulcer) being a stage with one of the most severe complications. VLUs are unfortunately frequently encountered, and wound recurrence often appears, too. Wound reopening after a period of completed epithelization of a previous venous ulcer due to exposure to causal factors and lack of prevention is considered to be the reason for recurrence of VLUs. Venous ulcers have a high recurrence rate that may increase through the years. Epidemiological evidence on their incidence and risk factors is scarce due to the lack of patient follow-up in outpatient clinics and adherence to treatment after healing. Common risk factors for VLU occurrence, such as the female gender, being elderly, and having obesity, also seem to be involved in recurrence occurrence. For example, obesity increases, by 8.7 times, the risk of recurrence [14]. Also, some comorbidities, such as systemic arterial hypertension, can bring an additional risk. According to our data, the main causes of recurrence are the fact that the venous reflux is not interrupted after the closure of the ulcer and/or the fact that the patients are not compliant with compressive therapy, but our study could not achieve a correlation of the recurrence with the type of bacteria identified at the first onset of the ulcer. The literature data demonstrate that the clinical approach to people with venous ulcers should not be finished when the wound is healed. For ulcer recurrence prevention, interventions addressing systemic factors, in addition to the topical management of the wound, are essential [14]. Although 80% of lower limb wounds develop as a result of venous insufficiency, other causes include arterial disease and diabetes [15]. With regard to this, a complete differential diagnosis should be performed.

Managing VLUs is quite controversial, complex, and challenging, particularly in cases involving recurrent ulcers. Currently, there is no standardized universal protocol regarding therapeutic management. The treatment of VLUs requires a multifaceted approach, focusing not only on wound care but also on addressing underlying causes, promoting healing, and preventing recurrence. Wound care is a fundamental aspect of VLU management. Proper wound dressing and regular cleaning are essential for preventing infection, facilitating healing, and promoting a moist wound environment. Because wound closure is often complicated by the presence of associated infections, we investigated the bacteria species involved in VLU infections and their sensitivity to antibiotic therapy. At the same time, we analyzed whether the microorganism species detected differed between recurrent VLUs and first-onset VLUs and whether the resistance of those bacteria to antibiotics increases. Understanding these infections’ microbial profiles and antibiotic susceptibility patterns is crucial for optimizing treatment strategies and improving patient outcomes.

The data related to the bacteria responsible for the VLU infection that we analyzed refer to the results obtained from the swab bacteriological cultures. Although there are studies that, for an accurate result, recommend swabbing after ultrasonic debridement and biopsy in the case of skin and soft tissue infection [16], there are strong literature data that claim that swabbing and deep-tissue cultures are equally reliable for the initial monitoring of antimicrobial treatment if the infection does not involve the bone [17,18,19]. Our analysis revealed several important findings regarding the bacteria species associated with VLU infections. All sample cultures from the recurrent VLU group were positive, and on the other side, 74% from the first-onset active VLU group were positive. *P. aeruginosa* was the most prevalent pathogen in both groups. This finding was consistent with previous studies that identified *P. aeruginosa* as a common culprit in chronic wound infections including VLUs [20,21]. However, according to our results, in the case of recurrent ulcers, *P. aeruginosa* was more frequently encountered compared to the first-onset active VLU group. Multiple drug-resistant *Pseudomonas* strains are a new threat because of their biofilm-forming ability, making them more potent and difficult to treat [22]. Their prevalence raises an alarm signal regarding the need for vigilance and targeted interventions to manage and control *P. aeruginosa* infections in VLU patients.

In addition to *P. aeruginosa*, other bacterial species were also found to be prevalent in VLU infections. These included other bacteria species usually encountered in wound infections, like Proteus mirabilis or Staphylococcus aureus, and other species like *Klebsiella* spp., *Escherichia coli*, *Enterococcus* sp., *Providencia rettgeri,* and *Acinetobacter baumannii*. The presence of diverse bacterial species highlights the polymicrobial nature of VLU infections and indicates that a multifaceted approach may be necessary for effective treatment. The assessment of antibiotic susceptibility profiles yielded crucial insights into the management of infected VLUs. The significant correlation observed for Amikacin and Meropenem suggests that these antibiotics may be particularly effective in cases where *Pseudomonas aeruginosa* is the predominant pathogen. Although our study, as well as other literature data [23], highlighted some antibiotics that can treat *Pseudomonasa*. infection, we must consider the fact that in case of colonization with multiple drug-resistant Pseudomonas strains, treatment consists of a combination of association of antibiotics with surgical debridement and repeated wound cleaning with antiseptic solutions and dressings [24]. Results from our study emphasize the seriousness of these cases, which are often resistant to antibiotic treatment.

However, our study also highlighted the issue of antibiotic resistance in VLU-associated infections. Several bacterial species showed resistance to various antibiotics, including Ceftriaxone, Cefazolin, Cefepime, Ciprofloxacin, Levofloxacin, Meropenem, Piperacillin, and Ticarcillin. The presence of antibiotic-resistant strains emphasizes the urgent need for judicious antibiotic use and the implementation of antibiotic stewardship programs. It is imperative to select appropriate antibiotics based on susceptibility testing to avoid treatment failures and the development of further resistance. Due to antimicrobial treatment that can last from 7–10 days to 4–6 weeks it should be considered to administer antibiotics locally, where possible, in order to reduce the risk of systemic toxicity (hepatotoxicity, nephrotoxicity, etc.)

The laboratory tests conducted in this study provided valuable insights into the association between nonspecific biomarkers and the presence of recurrent VLUs compared with first-onset VLUs. These tests allowed us to investigate potential correlations between various laboratory parameters and the severity of the condition, which is crucial for optimizing diagnostic and treatment strategies [25].

The analysis of blood cell counts and inflammatory markers revealed significant differences between the patients with active recurrent VLUs and the patients with first-onset active venous ulcers. Specifically, the recurrent VLU patients showed significantly lower levels of white blood cells (WBCs) compared to the first-onset active VLU patients. The significantly higher total count of WBCs and the predominance of neutrophils in the WBC differential in patients with recurrent VLUs suggest a less pronounced inflammatory response and potential impairment in the adaptive immune response in patients with recurrent chronic ulcers [26,27,28]. This observation also explains the predominant presence of infection with *Pseudomonas* species in patients with recurrent venous ulcers, as well as the higher frequency of polymicobial infections and even fungal infection. Fungal infections are also associated with worsening and delaying the healing process for chronic wounds, commonly involving poor clinical outcomes. The most frequently isolated pathogenic fungi and opportunistic pathogenic fungi mainly include Candida species and filamentous fungi [29]. Further investigation into the underlying mechanisms of this difference could provide valuable insights into the pathophysiology of recurrent VLUs and potentially lead to more targeted therapeutic interventions.

The recurrent VLU patients exhibited significantly higher levels of CRP and fibrinogen compared to the first-onset active VLU patients, suggesting a prolonged inflammatory response in patients with recurrent VLUs [30]. These findings support the notion that chronic inflammation may play a critical role in the recurrence of VLUs, and targeting inflammatory pathways could be a promising therapeutic avenue. The laboratory tests also included an assessment of glycemia and urea levels. Although no statistically significant differences were observed between the groups, it is essential to note that these parameters can provide valuable information about a patient’s general health status and potential comorbidities that may impact VLU healing and recurrence.

Among the muscle tissue markers assessed, CK levels were significantly higher in the study group compared to the control group. The elevated CK levels in the study group could indicate greater muscle damage or trauma, potentially contributing to the chronicity of VLUs. Understanding the relationship between muscle damage and VLU recurrence could lead to novel therapeutic approaches aimed at improving muscle function and mitigating ulcer recurrence [31]. The findings shed light on potential mechanisms contributing to VLU recurrence such as altered inflammatory responses, muscle damage, and chronic inflammation.

Our study shed light on the bacteria species involved in VLU infections and their antibiotic susceptibility patterns. *P. aeruginosa* emerges as a prominent pathogen, and its sensitivity to specific antibiotics provides valuable guidance for targeted therapy. However, antibiotic resistance remains a significant concern, necessitating cautious antibiotic use and robust infection control measures [32].

A range of dressings is available, including hydrocolloids, foam dressings, and alginate dressings, each offering unique properties to support wound healing [24,33]. However, effective VLU management goes beyond wound care alone. Addressing the underlying venous insufficiency is crucial to prevent further ulceration and recurrence [34]. In some cases, venous ablation procedures may be necessary to address the underlying venous reflux. Different treatment options to treat incompetent veins responsible for venous reflux are available. These procedures help restore normal venous circulation and reduce the risk of VLU recurrence [35]. However, because some studies concluded that at a five-year follow-up, a significantly higher varicose vein recurrence rate appeared after endovenous procedures compared to classic ones [36,37,38], surgical intervention remains a feasible strategy and should be recommended. In certain cases, especially when VLUs are complicated by tissue necrosis or infection, surgical debridement or tissue grafting can aid in wound healing and promote the formation of healthy granulation tissue and should be considered [39]. Considering the longer healing period of recurrent ulcers, as well as the high costs associated with this process, once the wound is closed, the prevention of recurrence is essential. In all the cases, we recommended venous reflux ablation once the wound is closed (phlebectomys, cryostripping or classic stripping, or foam sclerotherapy) and compression therapy. In cases where, due to intense local inflammatory phenomena, a surgical intervention would be contraindicated, compression therapy remains the only reliable long-term option for preventing recurrences [10,40,41,42]. A comprehensive understanding of the microbial profile in VLU infections is crucial for developing evidence-based approaches to optimize treatment strategies and improve patient outcomes. However, it is essential to acknowledge that this study’s limitations, such as its retrospective design and relatively small sample size, may warrant further investigation to validate and expand upon these findings. Future research in larger prospective cohorts could help solidify the clinical significance of these laboratory parameters in the context of VLU recurrence and provide a basis for personalized treatment strategies. Furthermore, our study did not control for the type-1 statistical error in the face of multiple comparisons. The generation of multiple *p*-values through various statistical tests increases the risk of the type-1 error, where a true null hypothesis is incorrectly rejected. This limitation is significant in studies with multiple endpoints or comparisons as it can lead to the overestimation of statistically significant findings. Future studies could benefit from applying correction methods, such as the Bonferroni correction or the False Discovery Rate (FDR), to adjust for the multiplicity of testing and reduce the likelihood of type-1 errors.

Additionally, the nature of our study population and the setting may limit the generalizability of our findings. Our study was conducted in a specific clinical setting with a defined patient population, which may not fully represent the broader population of individuals with VLUs. Differences in healthcare systems, patient demographics, and clinical practices could influence the applicability of our results to other settings.

Despite these limitations, we believe that our findings contribute to the existing knowledge on the microbiological aspects of VLUs and highlight the importance of tailored antibiotic therapy based on specific microbial profiles. Recognizing these limitations underscores the need for further prospective, multicentric studies with rigorous design to validate our findings, explore causality, and enhance the generalizability of the results to broader populations. Future research in this area should focus on larger prospective studies to further explore the interactions between microbial species, antibiotic susceptibility, and clinical outcomes in VLU management. Ultimately, this knowledge will contribute to the development of more effective and tailored interventions for patients with VLU-associated infections.

## 5. Conclusions

The identification of common and uncommon type of bacteria species involved in VLU infections, along with their sensitivity to antibiotics, highlights the importance of tailored antibiotic therapy for effective treatment. While antibiotic-resistant strains pose a significant challenge, antibiotic stewardship is of utmost importance to ensure the responsible use of antibiotics and preserve their effectiveness for future generations. *P. aeruginosa* was the most prevalent pathogen associated with wound infection in this study, but other bacteria, including species rarely encountered in wound infection like *Providencia rettgeri* and *Acinetobacter baumannii* or fungal infections (*Candida* sp.), were encountered, too. According to this study’s results, the following steps are recommended in curing infected VLUs: empiric antibiotic treatment should be categorically avoided, and antibiotic therapy should be used according to the antibiogram results.

## Figures and Tables

**Table 1:** Clinico-demographic characteristics of the patients.

Parameter	Recurrent VLU Group		First Onset Active VLU Group		*p*-Value
	N (%)	Mean ± SD	N (%)	Mean ± SD	
Women	57%	8.5 ± 7.18	85%	2.8 ± 2.98	0.005
Men	42%	4.8 ± 6.55	14%	2.2 ± 2.86	0.143
Age > 65 years	39%	73.4 ± 4.91	77%	68.3 ± 5.31	0.262
Native environment					
Urban	24%	3.6 ± 2.65	44%	4.1 ± 4.07	0.705
Rural	75%	8.7 ± 6.95	55%	5.1 ± 4.57	0.074
Comorbidities					
DM	39%	6.5 ± 5.27	44%	3.5 ± 3.27	0.048
CVDs	51%	7.5 ± 6.05	48%	3.555 ± 3.56	0.024
PAD	24%	4.1 ± 3.32	14%	1.9 ± 1.51	0.018
Antecedent of DVT	42%	6.6 ± 5.25	33%	3.7 ± 3.47	0.065
CKD	6%	0.8 ± 0.32	3%	0.7 ± 0.46	0.217
Obesity (BMI ≥ 30 kg/m^2^)	51%	6.4 ± 5.04	37%	3.5 ± 3.37	0.050

Legend: Diabetes mellitus (DM); deep-vein thrombosis (DVT); peripheral arterial disease (PAD); Cardiovascular Diseases (CVDs); Chronic Kidney Disease (CKD); mean ± standard deviation (SD).

**Table 2:** Bacterial infections according to the antibiogram/fungogram (sensitive, intermediate sensitive, resistant).

Infection	No. Study/Control Group	The Antibiotic Sensitivity Profiles			*p*-Value
		Sensitive	Intermediately Sensitive	Resistant	
*P. aeruginosa*	24/10	AK, CAZ, CEF, CIP, IPM, GM, LVX, MERO, PIP, TZP	S, CEF, IPM, LVX	CRO, CZ, CIP, LVX, MERO, PIP, TIC, TM	0.020
*Proteus mirabilis*	2/1	AK, AM, AMC, CEF, CZ, GM, IPM, SXT	IPM	-	0.042
*Enterococcus* sp.	½	AM, AMC, CM, E, GM, VA	CIP	-	0.054
*Staphylococcus aureus*	3/3	GM, CIP, CM, SXT	-	CXM, CM, OX	0.049
*Acinetobacter baumannii*	1/1	AK, CFP, IPM, MERO	-	CAZ, CEF, CIP	0.219
*Klebsiella* spp.	2/1	AK, AM, MERO, IPM, GM, PIP, TZP	GM	SXT	0.015
*Escherichia coli*	1/3	AK, AM, CRO, CEF, CIP, IPM, GM, PIP, TZP	-	LVX, SXT	0.243
*Providencia rettgeri*	1/0	AK, CAZ, CEF, CIP, IPM, GM	-	-	1.015
*Group B Streptococcus*	1/0	AM, AMC, CXM, GM, LVX	-	CM, E, SXT	0.354
*Candida albicans*	1/0	ECN, MIC, AMB, FCZ, NYS; VOR	-	5-FC	2.540
*Salmonella Paratyphi B*	1/0	AM, AMC, CRO, GM, LVX	-	CM, E	1.024
*Morganella morganii*	0/1	AK, CRO, CZ, CEF, CIP, IPM, GM, MERO, LVX, PIP	AMC	SXT	0.810

Amikacin = AK; Ceftazidime = CAZ; Ceftriaxone = CRO; Cefazolin = CZ; Cefepime = CEF; Cefuroxime = CXM; Cefoperazone = CFP; Ciprofloxacin = CIP; Imipenem = IPM; Gentamicin = GM; Levofloxacin = LVX; Meropenem = MERO; Piperacillin = PIP; Piperacillin+tazobactam = TZP; Streptomycin = S; Ticarcillin = TIC; Tobramycin = TM; Ampicillin = AM; Oxacillin = OX; Amoxicillin + clavulanic acid = AMC; Trimethoprim/sulfamethoxazole = SXT; Clindamycin = CM; Erythromycin = E; Vancomycin = VA; Econazole = ECN; Miconazole = MIC; Amphotericin B = AMB; Fluconazole = FCZ; Nystatin = NYS; Voriconazole = VOR; 5-Fluorocytosine = 5-FC.

**Table 3:** Values of different hematological and biochemical markers.

Laboratory Tests	Study Group Mean ± SD	Control Group Mean ± SD	*p*-Value
WBC (n)	6.4 ± 0.97	7.8 ± 2.34	0.008
RBC (n)	4.4 ± 0.50	4.4 ± 0.47	0.066
Neutrophils (%)	70.2 ± 8.27	64.2 ± 9.09	0.011
Lymphocytes (%)	17.9 ± 6.39	23.6 ± 8.02	0.003
CK (U/L)	147.6 ± 79.41	101.9 ± 83.02	0.035
CK-MB (U/L)	19.7 ± 4.55	22.0 ± 11.05	0.307
CRP (mg/L)	32.8 ± 19.07	16.2 ± 10.26	0.005
Fibrinogen (mg/L)	511.4 ± 120.59	435.8 ± 112.93	0.016
ESR (mm/h)	48.0 ± 11.34	31.0 ± 11.15	0.001
Glycemia (mg/dL)	145 ± 84.94	115.8± 35.40	0.084
Hba1c (%)	6.3 ± 1.19	6.5 ± 1.17	0.433
Creatinine (mg/dL)	0.9 ± 0.24	0.9 ± 0.31	0.527
Urea (mg/dL)	45.4 ± 13.76	41.3 ± 13.62	0.264

Normal ranges (laboratory reference values): RBC, 4.5–5.9 × 10^6^/μL; WBC, 4–9.5 × 10^3^/μL; neutrophil percentage, 45–70%; lymphocyte percentage, 20–40%; ESR, 0–15 mm/h; CRP, 0–10 mg/L; fibrinogen, 200–393 mg/dL; CK, 30–170 U/L; CK-MB, 0–16 U/L; glycemia, 74–106 mg/dL; Hba1c, normal range 4–5.6%, prediabetes, 5.7–6.4%, diabetes, >6.5%; creatinine, 0.7–1.3 mg/dL men and 0.6–1.1 mg/dL women; urea, 15–45 mg/dL.

## Data Availability

Data generated in this study may be requested from the corresponding author.
